# Analysis and reduction of eddy current effects induced by tesseral end zonal gradient coils in different collimator geometries for SPECT/MRI integration

**DOI:** 10.1186/2197-7364-2-S1-A51

**Published:** 2015-05-18

**Authors:** Amine M Samudi, Karen Van Audenhaege, Gunter Vermeeren, Emmeric Tanghe, Luc Martens, Roel Van Holen, Wout Joseph

**Affiliations:** 1 INTEC, Ghent University/iMinds, Ghent, Belgium; 2 ELIS, Ghent University/iMinds, Gent, Belgium

SPECT and MRI each have their respective advantages and limitations. Combining these two technologies in a synergistic manner allows researchers to exploit the strengths of both techniques but also result in disturbing eddy currents. In this paper, we studied the temporal variation of the induced magnetic field due to the transverse and longitudinal gradient coils, in a full-ring multi-pinhole collimator. We also investigated the effect of the ring geometry (hexagonal or pentagonal) on the resulting eddy currents and reduced the eddy currents by adding gaps between the collimators. We modeled x, y, and z-gradient coils and different arrangements of the SPECT collimators using FEKO. We arranged the collimators in pentagonal and hexagonal arrangements and we added gaps between the collimators in the pentagonal arrangement. The setup was simulated with a broadband simulation from 0 to 10 kHz with a step of 400 Hz to cover the frequency range of the gradient on-off switching (a sinusoidal ramp from 500 mT/m to 0 mT/m within 0.25 ms). The collimator design contains 20 loft-holes with 500-μm-diameter pinhole openings. The density of the collimator is equal to 17.31±0.10 g/cm^3^, and the conductivity equal to 108 nΩ.m. Simulations showed that the hexagonal geometry induces larger eddy currents. By adding relatively small gaps between the collimators (1.7 mm), the maximum value of the induced magnetic field is reduced by 50.6 % and 75.8 % for transverse and longitudinal gradient coils, respectively. As a result, the maximum value of the induced field is now less than 2 % of the applied gradient field.

