# Erratum to: Analysis and reduction of eddy current effects induced by tesseral end zonal gradient coils in different collimator geometries for SPECT/MRI integration

**DOI:** 10.1186/s40658-015-0128-x

**Published:** 2015-11-09

**Authors:** Amine M. Samoudi, Karen Van Audenhaege, Gunter Vermeeren, Emmeric Tanghe, Luc Martens, Roel Van Holen, Wout Joseph

**Affiliations:** INTEC, Ghent University/iMinds, Ghent, Belgium; ELIS, Ghent University/iMinds, Ghent, Belgium

## Erratum

Unfortunately, the original version of this article [1] contained an error. The author’s name, ‘Amine M Samoudi’, was spelt incorrectly. This can be found corrected in the author list above.
